# Levels of immunoglobulin isotypes in serum and respiratory samples of patients with chronic obstructive pulmonary disease: a systematic review and meta-analysis

**DOI:** 10.1186/s12931-026-03590-w

**Published:** 2026-02-25

**Authors:** Dana Unninayar, Oluwafemi Segun-Ige, Luana Leticia Teixeira Nunes Porto, Beverley Shea, Donald William Cameron, Juthaporn Cowan

**Affiliations:** 1 Department of Medicine, University of Ottawa, Ottawa, ON Canada; 2 Inflammation and Chronic Disease Program, Ottawa Hospital Research Institute, Ottawa, ON Canada; 3 School of Epidemiology and Public Health, University of Ottawa, Ottawa, ON Canada; 4 Division of Infectious Diseases, Department of Medicine, University of Ottawa, Ottawa, ON Canada

**Keywords:** COPD, Chronic Obstructive Pulmonary Disease, Immunoglobulins, Ig, IgG, IgM, IgA, Systematic review and meta-analysis

## Abstract

COPD is a disease of progressive and irreversible airflow obstruction, with exacerbations (AECOPD) often triggered by infection. Immunoglobulins play an important role in immune defense and immune regulation, however their role in COPD is poorly understood. Therefore, the purpose of our systematic review and meta-analysis was to assess serum, sputum, and bronchoalveolar lavage (BAL) levels of IgG, IgG subclasses, IgA, and IgM in COPD, and the association between immunoglobulins and key clinical outcomes.

EMBASE and Ovid MEDLINE were searched from inception to April 2024. Study screening and extraction were completed by two independent reviewers. Non-randomized studies assessing immunoglobulin levels were included. Data was analyzed using RevMan 5.

Our search identified 1897 studies; 36 were included in meta-analysis. Compared with healthy controls, individuals with COPD showed elevated secretory IgA levels (Standard Mean Difference (SMD) 1.68 [95% Confidence Interval (CI), 0.78, 2.59], *N* = 2). IgG2 was significantly lower in COPD compared to controls [SMD − 0.91 (-1.24, -0.58)]. There was no significant SMD in other IgG subclasses, serum IgG, IgM, or BAL IgA. Serum IgG and subclass levels did not differ between stable and AECOPD (SMD 0.10 [-0.61, 0.81]).

Compared to COPD with normal IgG, the COPD-low IgG group had increased risk of COPD admissions (OR 1.32 [95% CI, 1.11, 1.56]), lower FEV1/FVC % (SMD − 2.26[-3.3, -1.14] and FVC (SMD − 0.31[-0.58, -0.05]). Systemic steroid use trended higher in COPD-low IgG (OR 2.32[0.94, 5.72]). There was no difference in 1-year mortality.

In conclusion, serum IgG2 was lower in COPD and low IgG was associated with increased COPD-related admissions. These findings support further investigation of IgG as a potential biomarker and therapeutic target in COPD.

**Supplementary Information:**

The online version contains supplementary material available at 10.1186/s12931-026-03590-w.

## Background

Chronic obstructive pulmonary disease (COPD) is a disease of progressive and irreversible airflow obstruction characterized by chronic respiratory symptoms including dyspnea and cough. COPD is one of the leading causes of morbidity and mortality worldwide. The diagnostic criteria and classification of COPD have evolved over the years. The GOLD Criteria is the current standard used worldwide to diagnose COPD and requires the confirmation of post-bronchodilator FEV1/FVC < 70% predicted by spirometry to support evidence of irreversible airflow obstruction in the appropriate clinical context [1]. Acute exacerbations (AECOPD) are characterized by an acute increase in airway inflammation, gas trapping, and reduced expiratory flow resulting in increasing dyspnea and possibly hypoxia and hypercapnia. Each AECOPD has been shown to increase the rate of loss of lung function and contribute to an increase in morbidity and mortality in this population. Infection is the most common trigger of AECOPD. The most frequently implicated pathogens include *Haemophilus influenzae*,* Streptococcus pneumoniae*,* Moraxella catarrhalis*, and respiratory viruses such as rhinovirus, parainfluenza, coronavirus, and influenza [2].

Immunoglobulins, which are glycoprotein molecules produced by B cells, play an important role in both preventing infection and regulating inflammation and homeostasis in the lung parenchyma [3]. Serum, sputum, and bronchoalveolar (BAL) Ig levels in COPD as well as in comparison to otherwise healthy individuals have been examined previously, however have yielded heterogenous results. To date, there has been no comprehensive report examining the association between Ig levels and COPD or related outcomes in people with COPD. Understanding the difference between the systemic and local Ig levels of COPD patients and healthy individuals may further our understanding of COPD pathogenesis and support novel therapeutic approaches for the management of AECOPD. Therefore, the primary purpose of this systematic review is to evaluate and analyse immunoglobulin levels in the serum, sputum, and BAL of people with COPD, and compare these levels to otherwise healthy controls. Secondary outcomes assessed include examining how Ig levels among COPD correlate with key clinical outcomes.

## Methods

This systematic review and meta-analysis were conducted in accordance with the Preferred Reporting Items for Systematic Reviews and Meta-Analysis Protocols (PRISMA-P) guidelines (Supplemental Fig. 1) and Cochrane Handbook [3]. The study protocol was published prior to the initiation of screening and was registered with Prospero (Registration Number: CRD42020192220) [4].

### Search strategy

A comprehensive search strategy was designed with the assistance of a medical librarian and the use of the PRISMA-P checklist as guidance and is included as Supplemental Fig. 2. We searched Embase Classic and Embase (1947 to October 2024) and Ovid MEDLINE (1946 to October 2024). The reference section of each study was also searched for additional publications that were not included in the search results.

### Study screening and inclusion

Studies were then screened by two independent reviewers (DU and JC) using a web-based systematic review software, Covidence (Covidence, Melbourne, Australia) after completing initial pilot exercises to address any discrepancies in the application of the inclusion and exclusion criteria [5]. The screening process was divided into two phases. The first phase involved the screening of references by study title and abstract. Study titles without an associated abstract moved forward to full-text screening (second phase) unless it was clear to both reviewers that the study could be excluded. In the second phase, the full text was screened using pre-defined inclusion and exclusion criteria. At this stage, foreign language articles, except for French-language articles, were excluded given the lack of resources and accessibility of article translation. After the completion of each screening phase by both reviewers, conflicts were documented and reconciled. Inter-rater reliability between reviewers was evaluated using Cohen’s Kappa coefficient.

### Study eligibility criteria

We included studies that reported the levels of IgG, IgG subclasses, IgA, IgA subclasses and IgM in the serum, sputum and/or BAL of adults (> 17 years) with a diagnosis of COPD or chronic bronchitis. Studies that enrolled people with asthma-COPD overlap, COPD with concomitant primary immunodeficiency, or people with COPD who were already receiving immunoglobulin replacement therapy were excluded. In terms of the type of studies, we included non-randomised studies including case reports, case series, cross-sectional studies, as well as retrospective and prospective observational studies. Review studies and studies that were based solely on in-vitro experiments or animal models were excluded.

### Data capture

Data extraction was then conducted in duplicate by two independent reviewers (DU and OS) using a predesigned template created on Covidence [5]. The following data was extracted: information on study identification (authorship, publication year, funding, affiliated centres, journal), methodological approach (type of study, eligibility criteria, sample size, definitions of COPD or chronic bronchitis, cutoff values for low Ig levels), participant characteristics (age, sex, co-morbidities, smoking status), Ig levels (serum, sputum and BAL IgG, IgA, IgM; serum, sputum and BAL IgG and IgA subclasses), medication (inhaled corticosteroids, systemic corticosteroids, antimicrobials, bronchodilators), COPD severity (GOLD Stage), COPD status (stable or exacerbation at time of Ig measurement), and patient outcomes (rate of AECOPD per annum, rate of AECOPD hospitalizations, severity of AECOPD, requirement for oral or systemic steroids during exacerbations, and mortality). Any inconsistencies in data extraction were recorded and reconciled by a third-party reviewer (JC).

### Study outcomes and definitions

For the primary outcome, we determined mean immunoglobulin (IgG, IgM, and IgA) levels in the serum, sputum, and BAL samples of people with COPD. When possible, we compared Ig levels between healthy controls and people with COPD. The definition of COPD and/or chronic bronchitis varied between studies and is outlined in the results section. Controls were pooled from studies where controls were defined as healthy adults without underlying respiratory conditions. Methods of Ig measurement in each study were also compared.

For the secondary outcomes, we aimed to study the association between Ig and COPD severity (as defined by the GOLD criteria), FEV1% predicted, FEV1/FVC % predicted, FVC, incident rate of acute exacerbations of COPD per annum, timing of immunoglobulin measurement in relation to recurrent or current exacerbations, AECOPD-related admissions, use of systemic corticosteroids during exacerbations, and mortality rates.

### Study quality

The Risk Of Bias In Non-randomized Studies of Interventions (ROBINS-I) was used to assess the risk of bias (ROB) in non-randomized studies included in the review and was performed by two independent reviewers (DU and OS) [6]. Studies were scored in the context of seven domains per the ROBINS-I tool, namely baseline confounding, selection of participants, classification of interventions, deviation from intervention, missing data, measurement of outcomes, and selection of the reported result. Each domain was rated low, moderate or high risk of bias. There were no defined quality criteria for study exclusion.

### Study analysis

Microsoft Excel ^®^ was used to calculate combined means with standard deviation of serum IgG, IgG subclasses, IgA, and IgM levels, as well as BAL IgA. After consulting a statistician with expertise in systematic reviews, when a study only summarized data with median and interquartile ranges, the median was used as an estimate of the mean, and an estimated standard deviation was calculated by dividing the IQR by 1.35 [3]. It was not possible to estimate mean from geometric mean and therefore studies reporting only geometric means for which we were unable to obtain the raw data were not included in analysis. Review Manager 5 (RevMan 5) was used to calculate the mean difference of Ig levels with 95% confidence intervals (CI) between participants with COPD and controls, and between COPD participants in an acute exacerbation (AECOPD) compared to stable COPD. A random effects model was used to account for possible unobserved heterogeneity between studies. There was insufficient data to calculate summative and comparative data for sputum immunoglobulins, and BAL IgG, IgG subclasses, IgA subclasses and IgM levels.

RevMan 5 was also used to calculate the association between low IgG levels and COPD GOLD Stage, FEV1, FEV1/FVC, FVC, systemic steroid use, mortality, and rate of COPD-related admissions.

We conducted sensitivity analysis in order to account for possible clinical heterogeneity. We conducted sensitivity analyses by whether COPD was defined by spirometry or not defined by spirometry, as well as by a standard cutoff of IgG < 7 g/L. There was insufficient reported comorbidity data to conduct sensitivity analyses by comorbidity status. Statistical heterogeneity was assessed using the I^2^ statistic.

## Results

### Search results and study selection

**Fig. 1:**
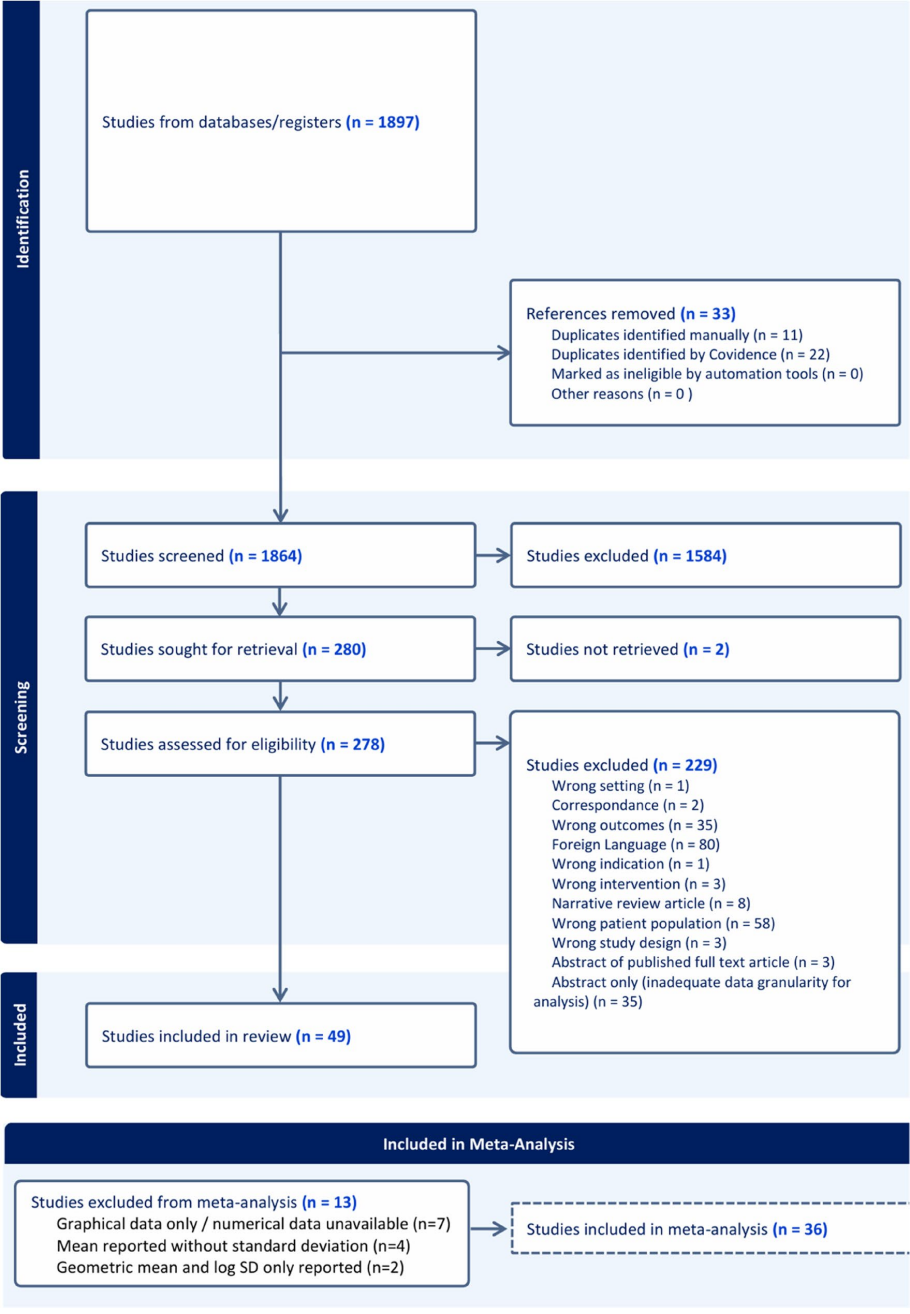
PRISMA Flow Chart. abbreviations: Preferred Reporting Items for Systematic Reviews and Meta-Analyses

We identified 1897 studies from our search, with 33 duplicates removed. 1864 studies were sought for retrieval however 2 studies could not be retrieved in spite of multiple requests through our interlibrary loan system. 278 studies were included for full text review and among these, 49 (with over 15,323 patients) met our pre-defined study criteria. The reasons for study exclusion were as follows: wrong patient population (*n* = 58), foreign language (*n* = 80), wrong outcomes (*n* = 35), abstract only with inadequate data granularity for analysis (*n* = 35), abstract of published full text article (*n* = 3), narrative review article (*n* = 8), correspondence (*n* = 2), wrong intervention (*n* = 3), wrong study design (*n* = 3), wrong indication (*n* = 1), and wrong setting (*n* = 1)(Fig. 1).

There was substantial agreement between reviewers during the abstract screening phase (Cohen’s Kappa = 0.6219), and fair agreement between reviewers during the full-text review phase (Cohen’s Kappa = 0.2183). Studies reporting variables that overlapped with those in one or more other studies were eligible for inclusion in the meta-analysis (*n* = 7022 patients, *N* = 36 studies). Thirteen studies were not included for meta-analysis for the following reasons: graph only or numerical data unavailable (*n* = 7), mean reported without standard deviation (*n* = 4), and geometric mean and log SD reported only (*n* = 2)(Fig. 1).

### Study characteristics and method of immunoglobulin measurement

Of the 49 studies included, there were 23 cross-sectional studies, 14 prospective studies, 6 retrospective studies, 3 randomized controlled trials (RCTs), and 3 post-hoc analyses of RCTs (Supplemental Table 1). The method of immunoglobulin measurement was described in 45/49 included studies, and included radial immunodiffusion (*n* = 17), ELISA (*n* = 8), immunofluorescence/flow cytometry (*n* = 5), both immunodiffusion and immunonephelometry (*n* = 1), and simple agar diffusion (*n* = 1) (Supplemental Table 1). The majority of studies included patients with spirometry-confirmed diagnoses of COPD however 5 did not specify the method of diagnosis, and 9 included patients with the diagnosis of chronic bronchitis by the American Thoracic Society (Supplemental Table 1). The majority of studies were conducted in the United States (17/49), 6/49 in Canada, 6/49 in China, 5/49 in India, and 15/49 in other countries (Supplemental Table 2).

Of the 49 studies included, 36 were amenable to meta-analysis. Some studies reported separate data for smokers and non-smokers, as well as frequent exacerbators (defined as ≥ 2 exacerbations per year) vs. infrequent exacerbators (defined ≤ 1 exacerbations per year) (Supplemental Table 1). In those instances, the estimated summative mean and standard deviation were calculated, and the summative value was used to perform the meta-analysis.

### Serum immunoglobulin levels in people with COPD

The mean (SD) IgG level was 9.84 (8.77) g/L in patients with COPD (*n* = 3927; *N* = 29 studies) and 18.05 (8.31) g/L in controls (*n* = 1001; *N* = 12) (Table 1). Among studies reporting IgG subclasses (*n* = 2190; *N* = 14), mean (SD) levels in COPD were: IgG1 = 5.07 (7.77) g/L, IgG2 = 2.69 (2.94) g/L, IgG3 = 0.59 (0.75) g/L, and IgG4 = 0.20 (0.82) g/L. Corresponding subclass levels in healthy controls (*n* = 167; *N* = 5) were: IgG1 = 7.95 (20.57) g/L, IgG2 = 3.59 (5.87) g/L, IgG3 = 0.82 (1.23) g/L, and IgG4 = 0.39 (0.77) g/L (Table 1).

**Table 1:** Mean (SD) immunoglobulin levels

Immunoglobulin	COPD (Mean (SD))	Controls (Mean (SD))
IgG	9.84 (8.77) (*n* = 3927, *N* = 29)	18.05 (8.31) (*n* = 1001, *N* = 12)
IgG1	5.07 (7.77) (*n* = 2190, *N* = 14)	7.95 (20.57) (*n* = 167, *N* = 5)
IgG2	2.69 (2.94) (*n* = 2190, *N* = 14)	3.58 (5.87) (*n* = 167, *N* = 5)
IgG3	0.59 (0.75) (*n* = 2190, *N* = 14)	0.82 (1.23) (*n* = 167, *N* = 5)
IgG4	0.20 (0.82) (*n* = 2190, *N* = 14)	0.39 (0.77) (*n* = 167, *N* = 5)
IgA	2.5 (1.4) (*n* = 2120, *N* = 28)	2.49 (0.89) (*n* = 1024, *N* = 13)
Secretory IgA	1.28 (0.69) (*n* = 85, *N* = 5)	0.069 (0.012) (*n* = 39, *N* = 2)
IgM	1.44 (4.81) (*n* = 703, *N* = 17)	1.48 (1.66) (*n* = 367, *N* = 10)

**Fig. 2:**
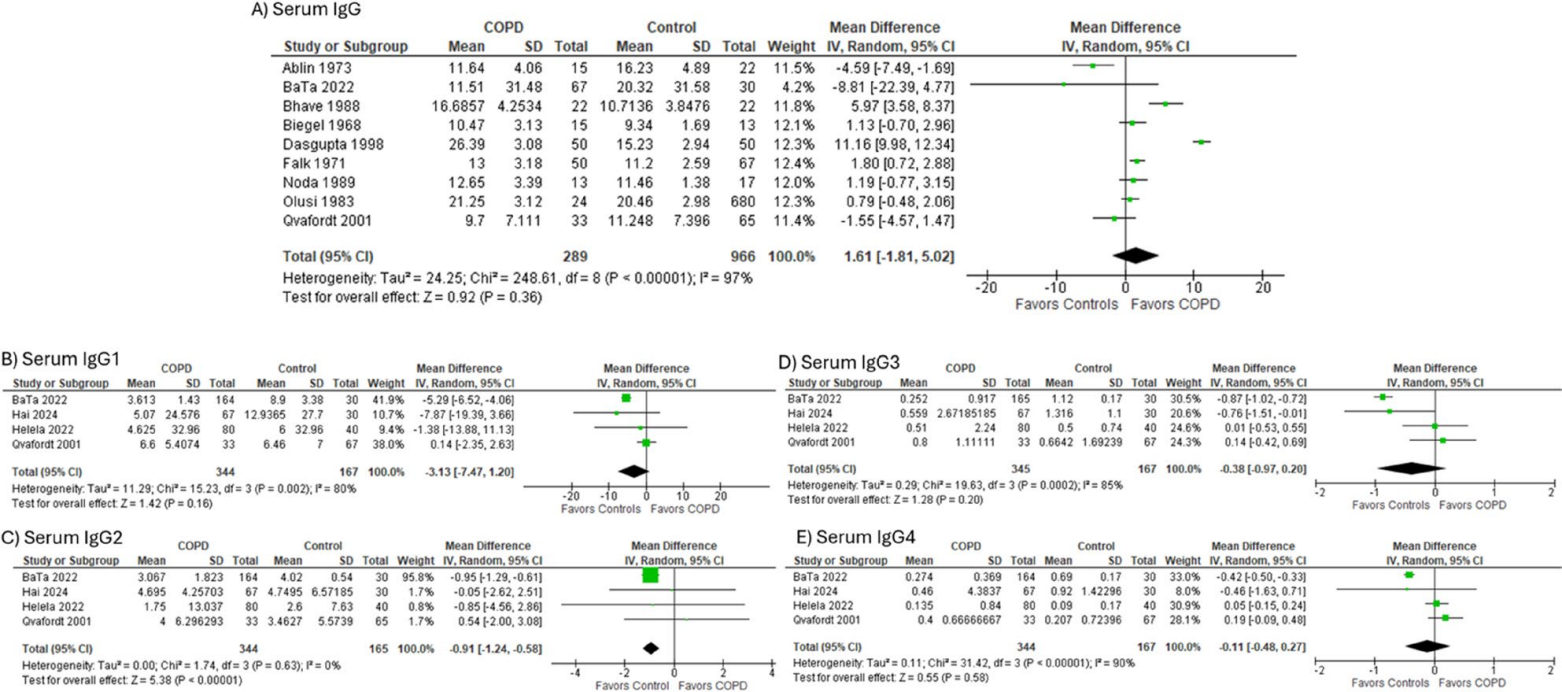
Immunoglobulin Levels in COPD compared to Controls. **A** Serum IgG levels in COPD vs. controls, (**B**) Serum IgG1 in COPD vs. controls, (**C**) Serum IgG2 Levels in COPD vs. controls, (**D**) Serum IgG3 levels in COPD vs. controls, (**E**) Serum IgG4 levels in COPD vs. controls. *Abbreviations*: *COPD* Chronic obstructive pulmonary disease, *Ig* Immunoglobulin, *SD* Standard deviation, *CI* Confidence interval

In the meta-analysis of studies directly comparing Ig levels between COPD and control participants (*n* = 289 vs. 966; *N* = 9), total IgG levels did not differ significantly [SMD = 1.61 (95% CI − 1.81 to 5.02)] (Fig. 2A; Table 2). Among IgG subclasses, only IgG2 was significantly lower in COPD compared to controls [SMD = − 0.91 (95% CI − 1.24 to − 0.58); *N* = 4] (Fig. 2B–E; Table 2).

**Table 2:** Immunoglobulin Levels in COPD and the association of low IgG levels and clinical outcomes

	*n* studies	SMD (95% CI)	OR (95% CI)	Heterogeneity (I^2^)
A) Immunoglobulins in COPD vs. Controls				
Serum IgG	9	1.61 (-1.81, 5.02)		97%
Serum IgG1	4	-3.13 (-7.47, 1.20)		80%
Serum IgG2	4	-0.91 (-1.24, -0.58)		0%
Serum IgG3	4	-0.38 (-0.97, 0.20)		85%
Serum IgG4	4	-0.11 (-0.48, 0.27)		90%
Serum IgM	8	-0.08 (-0.21, 0.05)		34%
Serum IgA	10	0.39 (-0.16, 0.94)		99%
Serum secretory IgA	2	1.68 (0.78, 2.59)		61%
BAL IgA	3	-0.00 (0.00, -0.00)		32%
B) Immunoglobulins in exacerbating vs. stable COPD				
Serum IgG	2	0.10 (-0.61, 0.81)		0%
Serum IgG1	2	0.16 (-0.24, 0.55)		0%
Serum IgG2	2	0.14 (-0.44, 0.73)		0%
Serum IgG3	2	-0.16 (-0.75, 0.44)		42%
Serum IgG4	2	-0.01 (-0.28, 0.27)		56%
C) Association between low serum IgG levels and clinical outcomes				
COPD admissions or readmissions	5		1.32 (1.11, 1.56)	39%
Number of COPD admissions in 1-year	2	1.30 (0.78, 1.81)		0%
Systemic Steroid Use	3		2.32 (0.94,5.72)	71%
1-year mortality	5		0.97 (0.49, 1.92)	61%
FEV1% predicted	5	-3.55 (-9.55, 2.50)		75%
FEV1/FVC% predicted	4	-2.26 (-3.37, -1.14)		0%
FVC	3	-0.31 (-0.58, -0.05)		82%
GOLD Stage III/IV vs. Stage I/II	2		0.77 (0.19, 3.04)	77%

*Abbreviations*: *COPD* Chronic obstructive pulmonary disease, *Ig* Immunoglobulin, *SMD* Standard Mean Difference, *CI* Confidence Interval, *OR* Odds Ratio, *FEV1* Forced expiratory volume in one second, *FVC* Forced Vital Capacity, *GOLD* Gold initiative for chronic obstructive lung disease

**Fig. 3:**
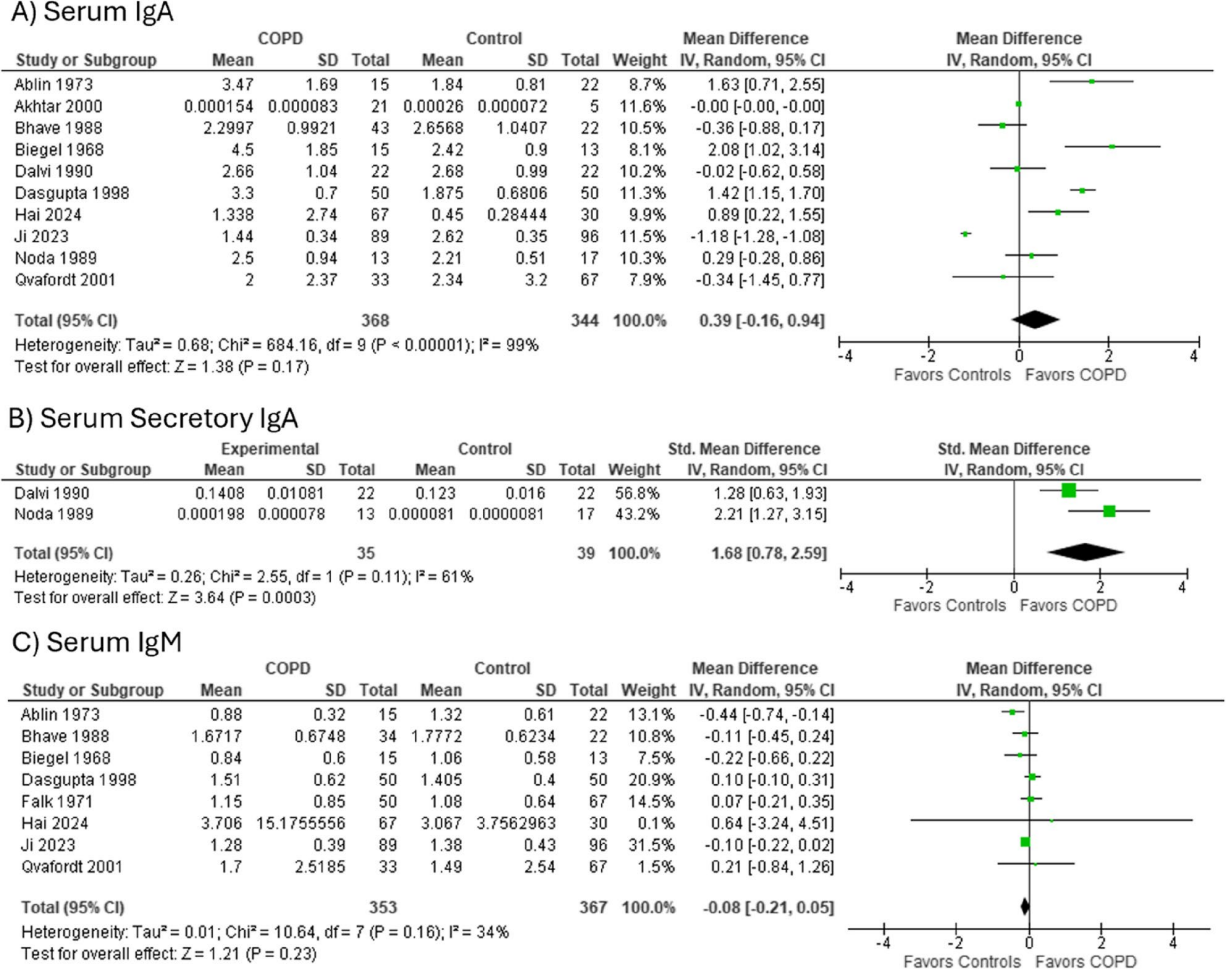
Immunoglobulin Levels in COPD compared to Controls. **A** Serum IgA levels in COPD vs. controls, (**B**) Serum Secretory IgA levels in COPD vs. controls, (**C**) Serum IgM Levels in COPD vs. controls. *Abbreviations*: *COPD* Chronic obstructive pulmonary disease, *Ig* Immunoglobulin, *SD* Standard deviation, *CI* Confidence interval

For IgA levels, mean (SD) were 2.5 (1.4) g/L for COPD (*n* = 2120, *N* = 28), and 2.49 (0.89) g/L for controls (*n* = 1024, *N* = 13). Mean (SD) secretory IgA levels were 1.28 (0.69) for COPD (*n* = 85, *N* = 5), and 0.069 (0.012) g/L for controls (*n* = 39, *N* = 2). On meta-analysis, total serum IgA levels were not significantly higher in COPD participants (SMD 0.39 [-0.16, 0.94]) (*N* = 10) but secretory IgA levels were significantly higher in COPD compared to controls (SMD 1.68 [0.78, 2.59], *N* = 2) (Fig. 3A and B; Table 2).

Serum IgM levels were 1.44 (4.81) g/L for COPD (*n* = 703, *N* = 17) compared to 1.48 (1.66) g/L for controls (*n* = 367, *N* = 10). On meta-analysis, the SMD in serum IgM comparing 353 people with COPD and 367 controls was − 0.08 [-0.21, 0.05] (*N* = 8) (Fig. 3C).

We conducted sensitivity analyses by assessing studies by the definition of COPD (with spirometry-confirmed diagnosis or reference to up-to-date guidelines) (Supplemental Table 1). IgG1 and IgG2 subclass levels were lower in the COPD group (SMD − 5.28 [-6.50, -4.06] and − 0.94 [-1.27, -0.60], respectively (Supplemental Fig. 3)).

### BAL or sputum immunoglobulin levels in people with COPD

BAL IgA in COPD was 0.007 (0.008) g/L (*n* = 48, *N* = 3) and in controls was 0.016 (0.012) g/L (*n* = 29, *N* = 3). BAL IgG in COPD patients was 0.0026 (0.0046) (*n* = 126, *N* = 2) while only 1 study reported a value of 0.038 (0.036769) g/L (*n* = 9) in controls. Sputum immunoglobulin levels were only reported in COPD (*n* = 69, *N* = 2) (Table 1).

There was insufficient control data to complete meta-analysis on sputum immunoglobulin levels.

### Serum immunoglobulin levels in stable COPD vs. during acute exacerbation

There was no difference in serum IgG levels or IgG subclass levels in people with acute exacerbations of COPD compared to those with stable disease (SMD 0.10 [-0.61, 0.81], *N* = 2) (Supplemental Fig. 4).

### Immunoglobulin levels and clinical outcomes

**Fig. 4:**
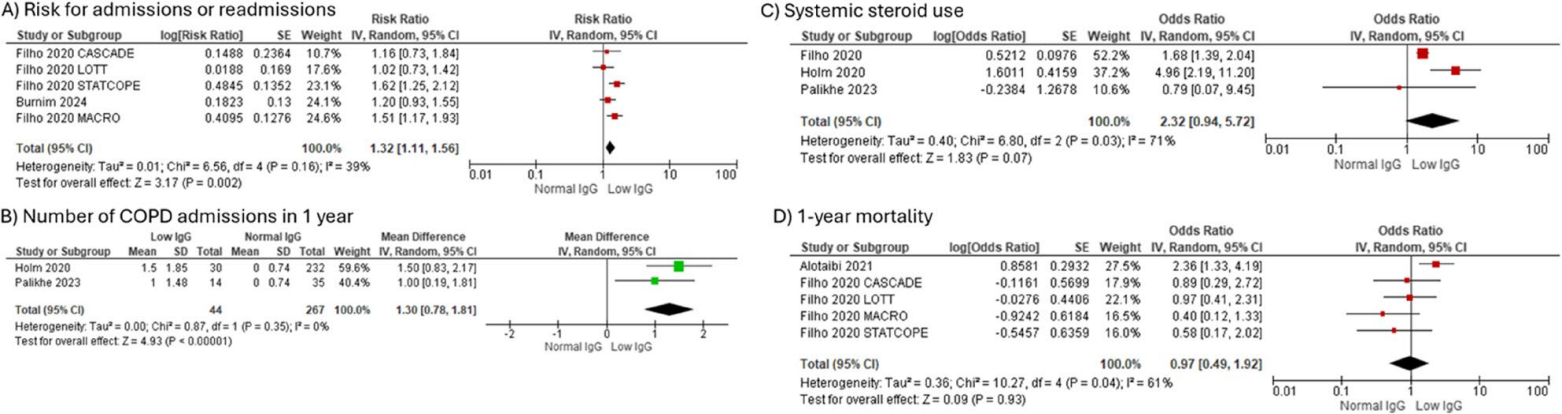
The association of low IgG levels with key clinical outcomes in COPD. **A** COPD admissions or readmissions in 1 year in low IgG-COPD group compared to normal IgG-COPD group, (**B**) Standard mean difference of number of COPD admissions in 1-year low IgG-COPD group compared to normal IgG-COPD group, (**C**) Systemic steroid use in low IgG-COPD group compared to normal IgG-COPD group, (**D**) 1-year mortality in low IgG-COPD group compared to normal IgG-COPD group. *Abbreviations*: *COPD* Chronic obstructive pulmonary disease, *Ig* Immunoglobulin, *SD* Standard deviation, *CI* Confidence interval

**Fig. 5:**
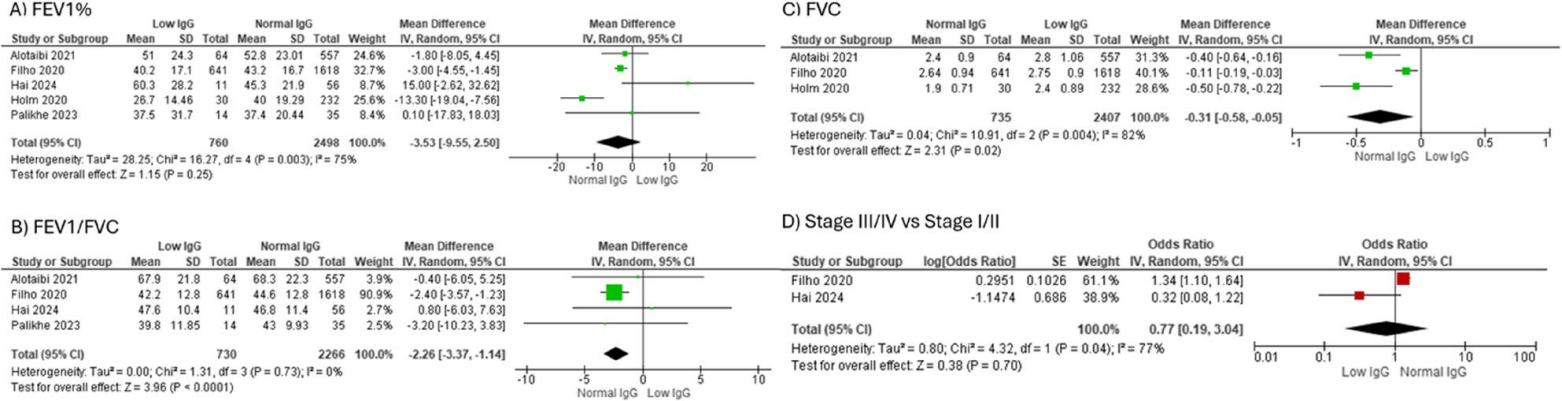
The association of low IgG levels with lung function in COPD. **A** Association of low IgG levels with FEV1% predicted in COPD, (**B**) Association of low IgG levels with FEV1/FVC % predicted in COPD, (**C**) Association of low IgG levels with FVC in COPD, (**D**) Odds of GOLD Stage III/IV vs. Stage I/II in low IgG levels vs. normal IgG. *Abbreviations*: *COPD* Chronic obstructive pulmonary disease, *Ig* Immunoglobulin, *SD* Standard deviation, *CI* Confidence interval

There was an increased risk of COPD-related admissions or readmissions in 1 year in the COPD-low IgG group compared to COPD with normal IgG (OR 1.32 [1.11, 1.56], *N* = 5) (Fig. 4A; Table 1). Additionally, COPD-low IgG group (*n* = 44) had a higher number of COPD admissions in 1 year compared to those with normal IgG (*n* = 267) (*N* = 2, SMD 1.30 [0.78, 1.81] (Fig. 4B). Low serum IgG did not have a significant association with incidence of systemic steroid use (OR 2.32 [0.94, 5.72], *N* = 3), (Fig. 4C). There was no significant difference in 1-year mortality or FEV1% between COPD-low IgG and those with normal IgG levels (Figs. 4D and 5A; Table 2, *N* = 5). Interestingly, FEV1/FVC % was significantly lower in COPD-low IgG group (*n* = 730) compared to those without low IgG (*n* = 2266) (SMD − 2.26 [-3.36, -1.14; *N* = 4], as was absolute FVC (SMD − 0.31 [-0.58, -0.05]; *N* = 3), COPD-low IgG *n* = 735, COPD with normal IgG *n* = 2407) (Fig. 5B, C). There was no difference in likelihood of GOLD Stage III/IV disease compared to Stage I/II in COPD-low IgG compared to COPD with normal IgG (Fig. 5D; Table 1, *N* = 2). There was insufficient data to assess association of IgG levels with total frequency of exacerbations either managed as outpatient or hospitalization.

Two studies assessed outcomes associated with low IgM. Hai et al. reported that there was no difference in IgM levels in those with acute exacerbations compared to stable COPD [7]. Palikhe et al. found that there was a significantly higher length of hospital stay (median (IQR)) in people with low IgM however there was no significant difference in the number ED visits, readmissions, AECOPD related admissions, systemic corticosteroid use, antibiotic use, FEV1%, and FEV1/FVC % [8].

Paul et al. was the only study to assess outcomes by low serum IgA and found that there was no difference in FEV1%, GOLD stage distribution, steroid use, and exacerbation frequency and history in people with low IgA compared to normal IgA [9].

We conducted sensitivity analyses by assessing studies based on the definition of COPD (with spirometry-confirmed diagnosis or reference to up-to-date guidelines) (Supplemental Table 1). FEV1% (SMD [95% CI] -5.30 [-11.1, 0.41]) and FEV1/FVC % (-2.34 [-3.47, -1.21]) were significantly lower in COPD-low IgG level group compared to controls (Supplemental Table 3, Supplemental Fig. 5) [7].

### Risk of bias assessment and heterogeneity

All included studies were assessed as having at least moderate risk of bias in one or more quality domains (Supplemental Table 4).

The majority (6/8) of pooled meta-analyses of Ig levels in COPD compared to controls demonstrated moderate to high heterogeneity [I^2^ 61–99%], while those comparing Ig levels in stable COPD compared to acute exacerbation of COPD had mild to moderate heterogeneity [I^2^ 0–54%]. Meta-analyses of low immunoglobulin levels and key clinical outcomes demonstrated variable heterogeneity with I^2^ ranging from 0% to 82%.

## Discussion

We conducted a systematic review and meta-analysis involving 49 studies with 7022 people with COPD. Compared to healthy controls, IgG2 subclass level was lower in COPD whereas there were no significant differences in serum IgG, other IgG subclasses, or IgM. Secretory IgA levels were significantly higher in COPD. Serum IgG and subclass levels did not differ between stable and AECOPD. Notably, low serum IgG level was significantly associated with COPD-related admissions and lower lung function (FEV1/FVC, FVC). It showed a non-significant trend toward increased systemic steroid use, but was not associated with increased mortality.

Immunoglobulins play an important role in regulating inflammation and mounting a response against pathogens in the lung parenchyma. IgA is the principal isotype in secretions of the epithelial lining of the respiratory tracts [10]. We found that secretory IgA (dimeric IgA with secretory component) levels were higher in COPD compared to healthy controls. A similar result was also found with total IgA. It has been postulated that upregulation of IgA occurs in severe COPD as an attempt to counteract abnormal host-microbial interactions and/or ongoing inflammation [11]. However, secretory IgA levels found in BAL are not elevated and, in our study, trended lower. pIgR, a receptor expressed by bronchial epithelial cells that internalizes IgA to transport to the lumen, has been shown to be downregulated in COPD [12]. It has been postulated that this defective transport mechanism could explain the discrepancy in serum and BAL IgA.

IgG opsonizes pathogens for phagocyte engulfment and activates complement [13]. There are four IgG subclasses that play different roles in the immune response and defence against infection. IgG1 is present in the highest amount and protects the body from protein-derived antigens, while IgG2 is responsible for polysaccharide responses. IgG3 and IgG4 are important in fighting viral respiratory infections [14]. Prior studies have found a higher incidence of total IgG and IgG2 subclass deficiency in COPD compared to healthy controls [15]. In our systematic review, we similarly found that IgG2 is significantly lower in COPD compared to healthy controls. Interestingly, Karnak et al. found that IgG in COPD was elevated compared to in their controls, while they found low IgG2 in lung tissue. It has been hypothesized that low IgG2 predisposes individuals to infections with encapsulated organisms. Given that control participants in Karnak were referred for bronchoscopy for chronic respiratory symptoms, it is possible that these individuals at baseline had lower IgG2 than otherwise healthy individuals, and therefore it is difficult to draw conclusions about COPD Ig levels from their study [16]. However, in our systematic review, there was no significant difference in IgG and other IgG subclasses between COPD and controls. In the meta-analysis of the limited evaluable studies, pooled estimates showed a trend toward higher levels of IgG in people with COPD. In contrast, when examining the mean IgG values reported across all individual studies descriptively (without pooling), the overall trend suggested lower IgG levels in COPD patients compared to controls (Supplemental Table 3). Notably, the control group had a relatively high mean IgG level with wide variance, suggesting a skewed distribution, which may have influenced the comparison and exaggerated the apparent difference between groups. Additionally, sensitivity analyses excluding studies with non-spirometry confirmed COPD also showed lower IgG subclasses in the COPD group (Supplemental Fig. 3). This discrepancy limits our ability to determine the true direction of serum IgG, IgG1, IgG3, and IgG4 changes in individuals with COPD. Given the high heterogeneity among the included studies and the consistent trend of lower IgG subclasses in COPD, the pooled data are more likely to reflect the true underlying pattern.

Among those with COPD, IgG3, but not IgG2, trended lower during an acute exacerbation compared to stable disease in meta-analysis. Prior studies have found that lower IgG2 and IgG3 subclass levels may predispose individuals to exacerbations. IgG2 deficiency would predispose individuals to bacterial-triggered AECOPD, however it is possible that the majority of people included in our analysis had viral infections, and therefore it could be expected that lower IgG3 would predispose these individuals to exacerbation. However, the pathophysiology of immunoglobulins in COPD is poorly understood at this time, and serum levels may not necessarily reflect sequestration of immunoglobulins in the airways and the dynamic regulation of immunoglobulins at mucosal sites during an exacerbation [17].

In our meta-analyses, low serum IgG level was associated with COPD-related admissions and potentially associated with systemic steroid use. By the GOLD guidelines, people admitted to the hospital with AECOPD should receive systemic steroids [1]. Unfortunately, the majority of studies reporting associations of low IgG levels with systemic steroid use and exacerbations are retrospective, with no temporal description of timing of steroid administration relative to the time of blood sample collection for Ig measurement. Given that in this population, oral steroids are a mainstay of treatment for an exacerbation of COPD, it is difficult to know whether underlying low IgG predisposes these patients to recurrent exacerbations, or whether certain individuals are predisposed to frequent exacerbations irrespective of IgG levels and develop low serum IgG levels secondary to frequent steroid requirements. Assessment of specific antibody production by measuring polysaccharide vaccine response at least 6 months after their last steroid treatment may help better identify those with an underlying antibody deficiency. However, this may not be practical for frequent exacerbators. Flow cytometric assessment of memory B cells in this population has also been proposed to help differentiate between low IgG levels from systemic steroid use compared to an intrinsic antibody deficiency [1].

This study has several strengths. It is the first systematic review and meta-analysis conducted to evaluate immunoglobulin levels in people with COPD, as well as to summarise current evidence on how low serum IgG levels correlates with key clinical outcomes. We conducted predefined sensitivity analyses, including stratification by spirometry-confirmed COPD diagnoses and by clinical definition of hypogammaglobulinemia or low IgG levels to enhance the reliability of our results. Our adherence to the PRISMA and Cochrane guidelines and independent screening, extraction, and analysis by two independent reviewers minimised the risk of reviewer bias.

However, our study also has several important limitations. First, while we used a random-effects model to account for variability in effect estimates, differences in COPD diagnostic criteria, patient characteristics, methods of immunoglobulin quantification may have contributed to the observed heterogeneity. Second, the accuracy of COPD diagnosis across the included studies was not consistently verified, raising the possibility of diagnostic misclassification. Third, there was inconsistency in the reporting of summary statistics, requiring us to estimate means and SDs from medians and IQRs, respectively. This introduces potential statistical imprecision, although the approach we followed is an accepted convention for meta-analysis. Some studies reported only geometric means with standard errors and therefore excluded from meta-analysis. Fourth, due to resource limitations and limited translation access, we were unable to include studies published in languages other than English or French. Finally, there were insufficient data to conduct a meta-analysis examining the relationship between Ig levels and exacerbation frequency in COPD.

## Conclusion

In conclusion, COPD had significantly lower IgG2 and higher secretory IgA levels compared to healthy controls. Our systematic review highlights that low IgG level is associated with increased risk of COPD-related admissions, lower lung function, and potentially systemic steroid use. Further research is needed to determine whether this association is causal or consequential, and to explore its potential utility as a predictive biomarker. Additionally, therapeutic trials evaluating the correction of low IgG or IgG2 levels in selected COPD populations could offer valuable insights into immune-targeted interventions.

## Supplementary Information

Supplementary Material 1.

## Data Availability

The data that support the findings of this study are available from the corresponding author upon reasonable request.
