# Supplementary material for: Levels of immunoglobulin isotypes in serum and respiratory samples of patients with chronic obstructive pulmonary disease: a systematic review and meta-analysis
Source: Respir Res. 2026 Feb 25;27:151. doi: 10.1186/s12931-026-03590-w (PMC13041067; doi:10.1186/s12931-026-03590-w)
Supplement: Supplementary file 1 — Supplementary Material 1. [file 12931_2026_3590_MOESM1_ESM.docx]

**Supplemental Figures and Tables**

Supplemental Figure 1. PRISMA-P Checklist
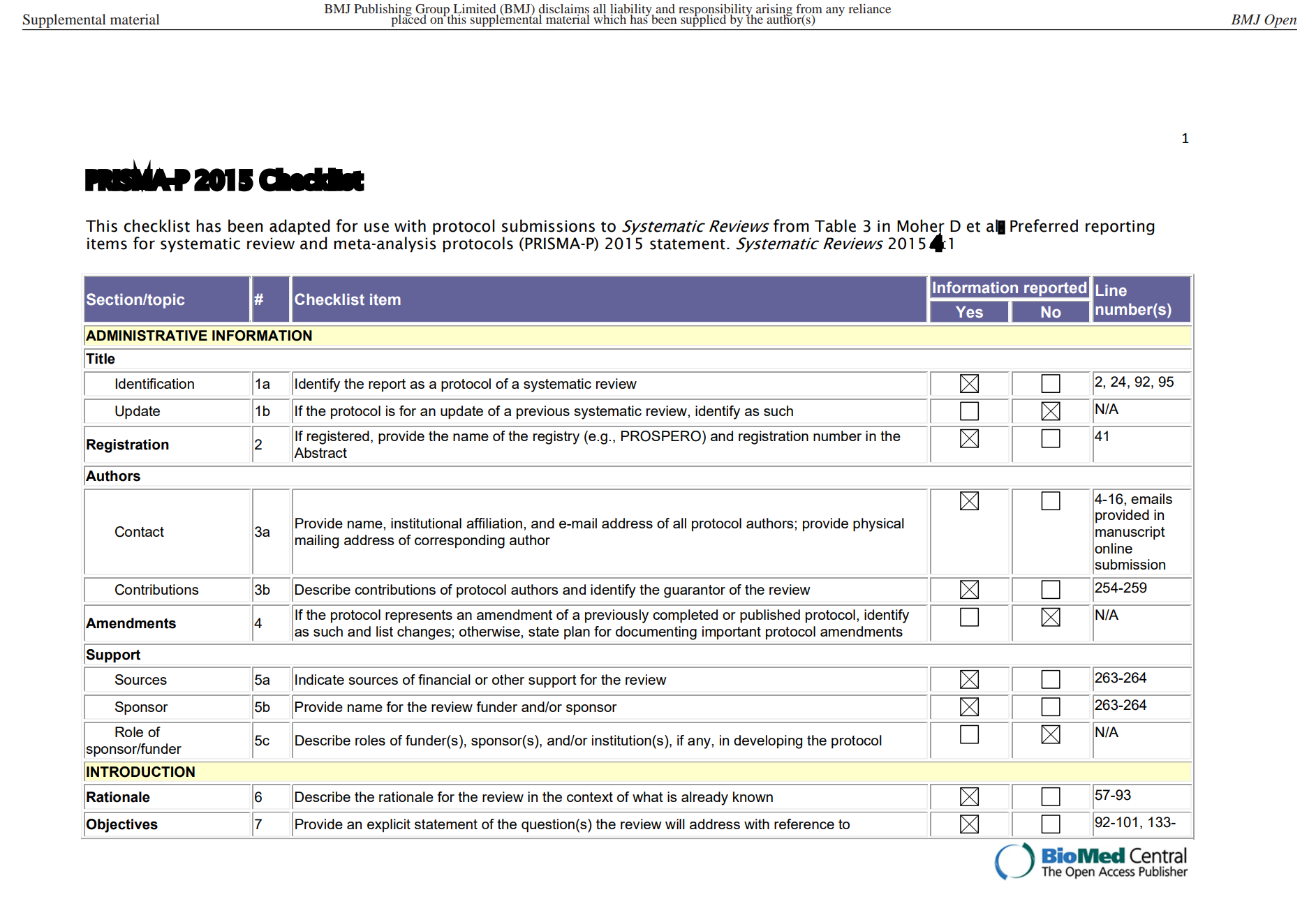

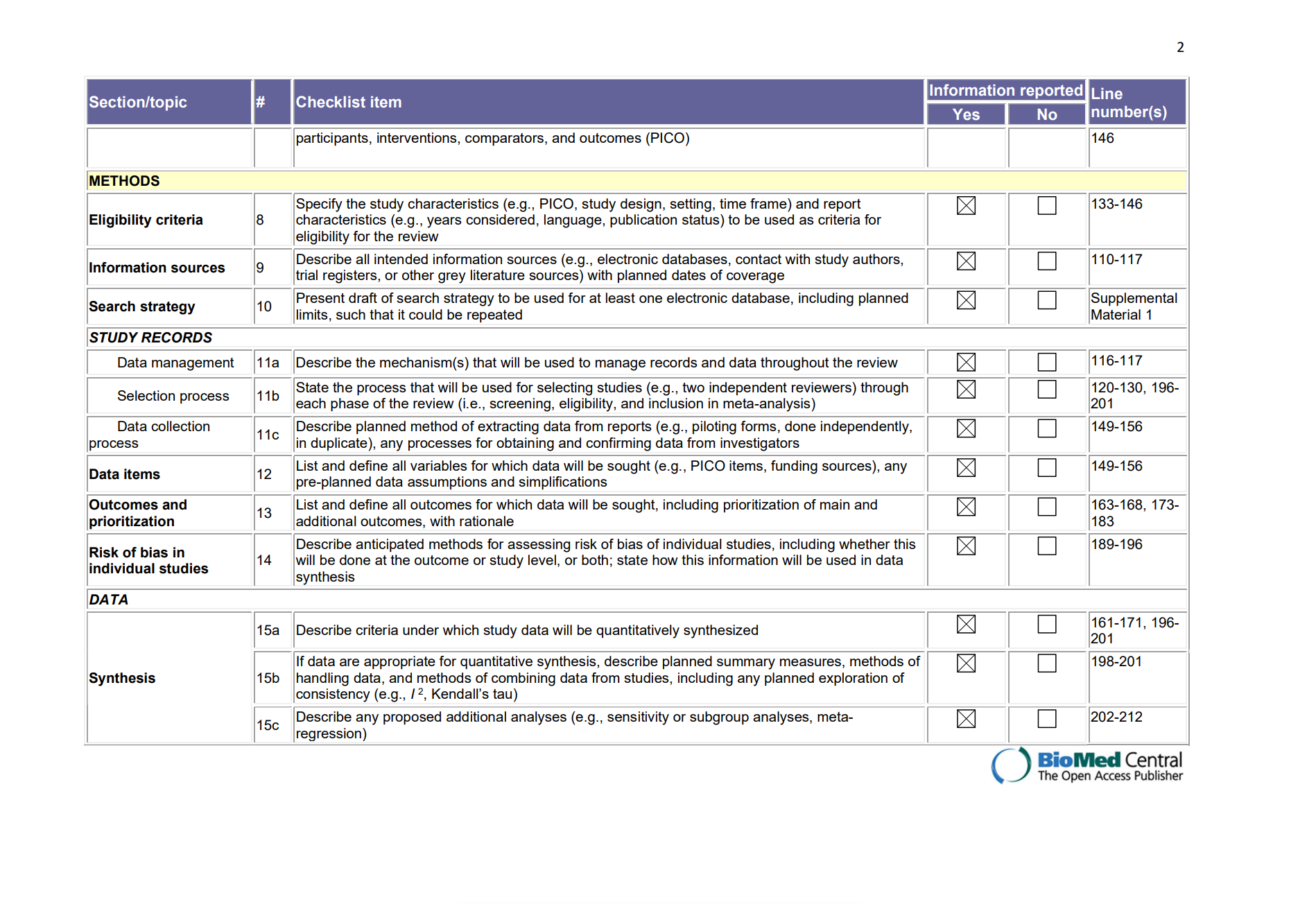

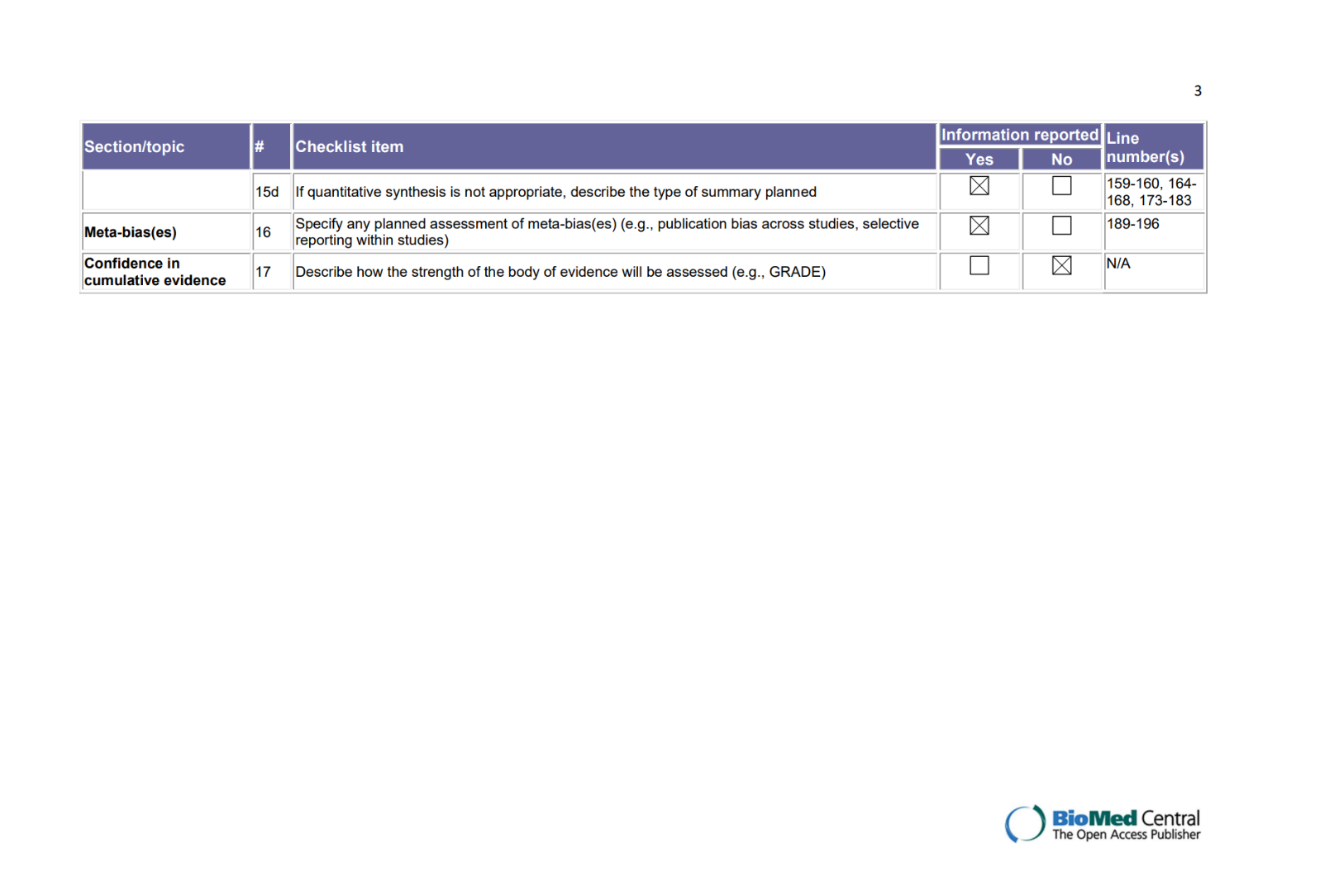


**Supplemental Figure 2.** Search Strategy


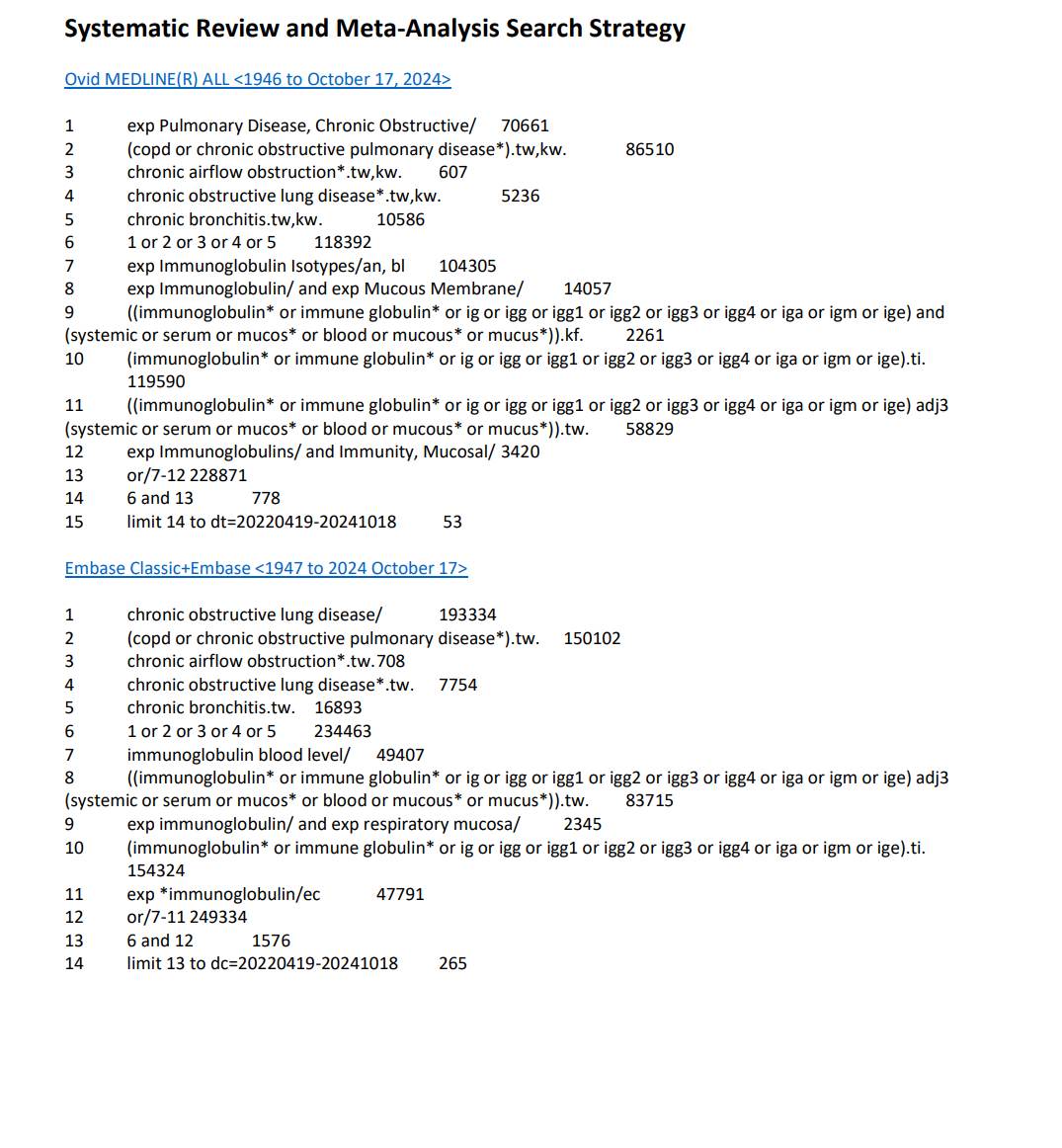


**Supplemental Figure 3**. Sensitivity analyses of studies with spirometry confirmed COPD diagnosis
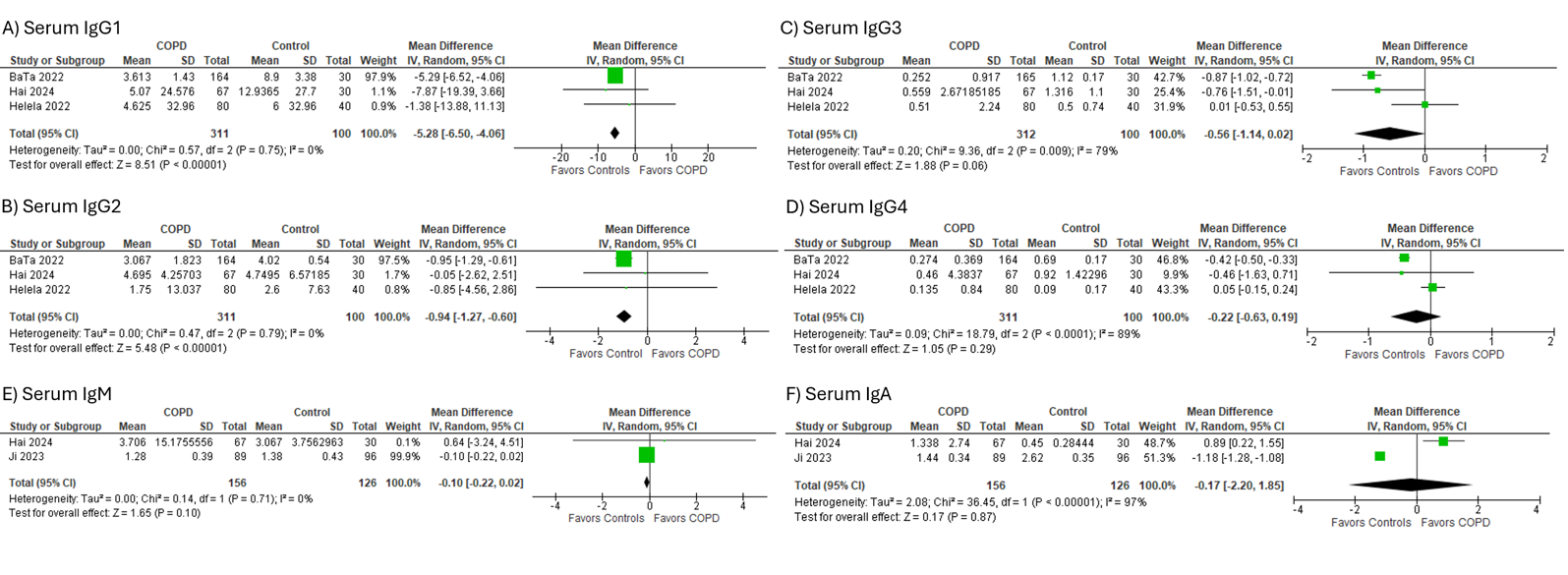


**Supplemental Figure 4.** Serum immunoglobulin levels in stable COPD vs during acute exacerbation


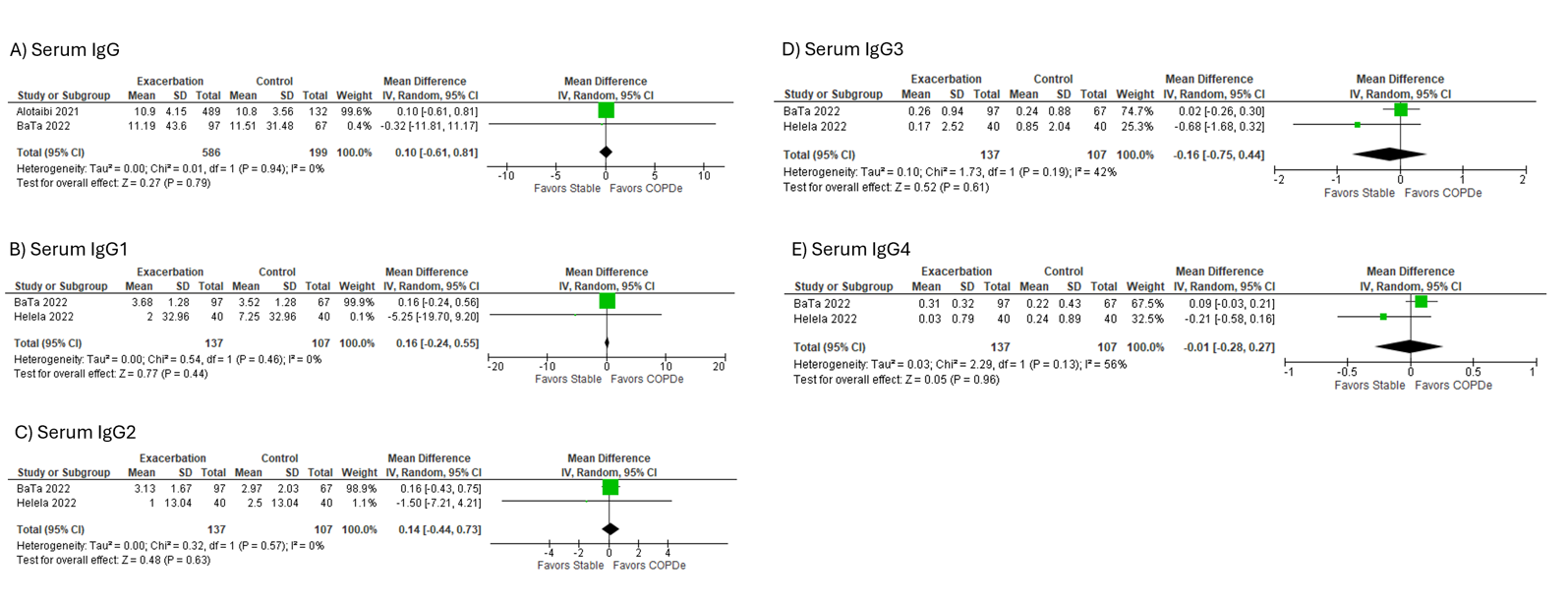


**Supplemental Figure 5.** Sensitivity analyses of studies with spirometry confirmed COPD diagnosis


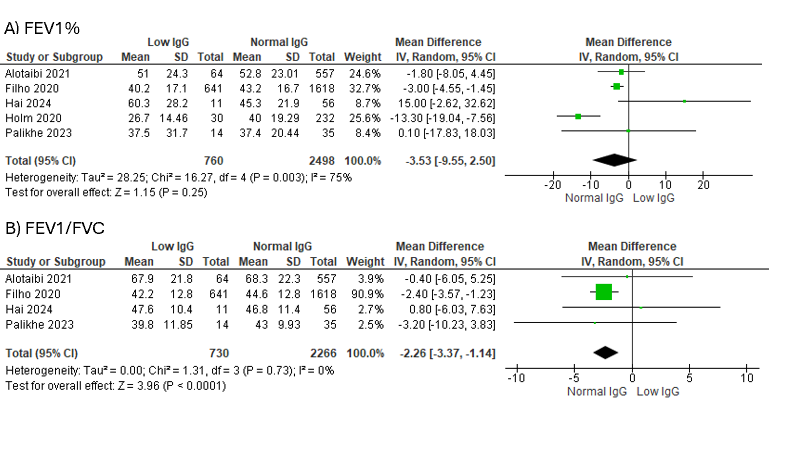


**Supplemental Table 1**. Characteristics of Included Studies

| Author, Year | Study Design | Number of COPD Patients | Number of controls | | Values Reported | | Comparator | | Diagnosis of COPD |
| --- | --- | --- | --- | --- | --- | --- | --- | --- | --- |
| Ablin, 1973 | CS, SC | 15 | 22 | | Serum IgG, IgA, IgM via indirect immunofluorescence | | Healthy controls | | Diagnosed with pulmonary emphysema based on Diagnostic Standards of Nontuberculosis Respiratory Diseases of the American Thoracic Society |
| Akhtar, 2000 | CS , SC | 21 | 5 | | Serum IgA and BAL IgA by radial immunodiffusion | | Healthy controls | | Severe chronic airway obstruction - FEV1 /FVC < 50% and absence of other significant disease that could alter IgA |
| Alotaibi, 2021 | P, SC | 621 | N/A | | Serum IgG by liquid chromatography–tandem mass spectrometry | | Exacerbation vs Stable | | Unspecified, recruited from COPD clinic |
| BaTa, 2022 | P, SC | 97 | 30 | | Serum IgG, IgG1, IgG2, IgG3, IgG4 by immunofluorescence technique | | Healthy controls | | Post-bronchodilator FEV1/FVC < 70% |
| Berber, 1995 | CS, SC | 21 | 18 | | IgA by nephelometry (Behring turbitimer) | | Healthy controls | | Not specified |
| Bhave, 1988 | CS, SC | 43 | 22 | | Serum IgG, IgM, IgA by single radial immunodiffusion method (Macini et al) | | Healthy controls | | Not specified (chronic bronchitis) |
| Biegel, 1968 | CS, SC | 15 | 13 | | Serum IgG, IgM, IgA by radial diffusion | | Healthy controls | | Not specified (chronic obstructive pulmonary emphysema clinical diagnosis) |
| Burnim, 2024 | AD, MC | 979 | 421 | | Serum IgG, IgG1, 2, 3, 4 by ELISA | | Controls | | FEV1/FVC < 70% |
| *Cass, 2021** | CS, SC | 44 | 45 | | Serum and sputum IgG, IgM, IgA by ELISA | | Healthy controls | | FEV1/FVC < 70% with bronchodilator reversibility < 12% and postbronchodilator < FEV1 80% |
| Chauhan, 1990 | CS, SC | 30 | 30 | | Serum and sputum IgG, IgM, IgA by single radial immunodiffusion | | Healthy controls, type A vs type B COPD | | FEV1< 75% with no significant improvement with parenteral bronchodilators |
| **Cvoriscec 1989*** | RCT, SC | 52 |  | | Serum IgA by laser nephelometry and radial immunodiffusion | | n/A | | Not specified (chronic bronchitis) |
| Dalvi **1990** | RCT, SC | 22 | 22 | | Serum IgA, Secretory IgA by single radial immunodiffusion | | Healthy controls | | Not specified (chronic bronchitis) |
| Dasgupta *1998* | CS, Sc | 50 | 50 | | Serum IgA, IgG, IgM by single radial diffusion | | Healthy controls | | Chronic bronchitis – persistent cough and sputum for 3 months in at least 2 consecutive years, 1 period of illness which had kept pt away from normal activities for at least 3 months, no evidence of pulmonary TB or bronchiectasis |
| Du, 2015 | CS, SC | 54 | 26 | | BAL IgA by ELISA | | Healthy controls | | GOLD criteria |
| **Falk, 1972** | CS, SC | 6 | 6 | | BAL and serum IgA, IgG by radial immunodiffusion | | Healthy controls | | FEV1/FVC < 50% |
| **Falk, 1971** | P, SC | 50 | 67 | | Serum IgA, IgG, IgM by radial immunodiffusion (Mancini method) | | Healthy controls | | FEV1/FVC < 75% |
| **Hai 2024** | P, SC | 67 |  | | Serum IgG, IgA, IgM, IgG1, 2, 3, 4 by immunofluorescence technique | | Healthy controls | | FEV1/FVC < 70% |
| **Harbitz 1980*** | CS, SC | 16 | - | | Serum and sputum IgA, IgM, IgG by immunodiffusion (Mancini) | | None | | Chronic bronchitis |
| He 2023 | P, SC | 155 |  | | Serum IgA, IgG, IgM measured by Sysmex XE-5000 hematology analyzer | | NIV | | FEV1/FVC < 70% |
| Helala, 2022 | R, SC | 80 | 40 | | Serum IgG1, 2, 3, 4 by immunophelometry | | Healthy controls, exacerbation vs stable | | COPD, unspecified |
| Holm, 2020 | P, SC | 262 | - | | Serum IgG, IgA, IgM, quantified by turbidimetry on a Roche Modular P instrumen | | Hypogammaglobulinemia vs non-hypogammaglobulinemia | | GOLD Criteria |
| Ho 2024 | P, SC | 53 |  | | Sputum IgA, IgM, IgE, IgG, IgG1, 2, 3, 4 | |  | | Not specified |
| Ji 2023 | R, SC | 89 AECOPD | 96 | | Serum IgM, IgA by immunoturbidimetry | |  | | GOLD classification |
| Karnak, 2001 | CC, SC | 24 | 17 | | Serum IgG, IgA, IgM, IgG1, IgG2, IgG3, IgG4 by nephelometry | | Healthy controls | | COPD by ATS definition |
| *Klaustermeyer, 1992* | CC, SC | 15 | - | | Serum IgG, IgA, IgM, IgG1, 2, 3, 4 by radial immunodiffusion | |  | | COPD by ATS (<15% change in FEV1 after bronchodilator therapy, history of >25 pack year tobacco use, and beta against-theophylline and corticosteroids in a dose of at least 10mg daily for longer than one year |
| Lee, 2022 | P, MC | 489 | - | | Serum IgG1, 2, 3, 4 by liquid chromatography-tandem mass spectrometry | | Smoking vs non-smoking | | COPD, not specified |
| Leitao Filho MACRO, 2018 | P, MC | 976 |  | | Serum IgG by immunonephelometry | |  | | GOLD, post-bronchodilator FEV1/FVC <70% |
| Leitao Filho STATCOPE, 2018 | P, MC | 653 |  | | Serum IgG by immunonephelometry | |  | | GOLD, post-bronchodilator FEV1/FVC <70% |
| Leitao Filho LOTT, 2018 | P, MC | 354 |  | | Serum IgG by liquid chromatography-tandem mass spectrometry | |  | | GOLD, post-bronchodilator FEV1/FVC <70% |
| Leitao Filho CASCADE,2018 | P, MC | 276 |  | | Serum IgG by liquid chromatography-tandem mass spectrometry | |  | | GOLD, post-bronchodilator FEV1/FVC <70% |
| Leitao Filho MACRO, 2020 | P, MC | 976 |  | | Serum IgG1, 2, 3, 4 by immunonephelometry | |  | | GOLD, post-bronchodilator FEV1/FVC <70% |
| Leitao Filho STATCOPE, 2020 | P, MC | 653 |  | | Serum IgG1, 2, 3, 4 by immunonephelometry | |  | | GOLD, post-bronchodilator FEV1/FVC <70% |
| Leitao Filho LOTT, 2020 | P, MC | 354 |  | | Serum IgG1, 2, 3, 4 by liquid chromatography-tandem mass spectrometry | |  | | GOLD, post-bronchodilator FEV1/FVC <70% |
| Leitao Filho CASCADE,2020 | P, MC | 276 |  | | Serum IgG1, 2, 3, 4 by liquid chromatography-tandem mass spectrometry | |  | | GOLD, post-bronchodilator FEV1/FVC <70% |
| Li, 2006 | P, SC | 62 |  | | Serum IgA, IgM, IgG by simple agar diffusion | |  | | COPD, unspecified |
| *Liu 2020* | 20 | 10 |  | | Tissue sIgA and dIgA | | Healthy controls | | FEV1/FVC < 70% and FEV1% predicted > 50% |
| *McCullagh, 2017* | P, SC | 29 |  | | Serum IgG, IgA, IgM, and IgE by nephelometry | |  | | FEV1/FVC < 70% |
| *McQuiston , 2022** | CS, MC | 32 |  | | Serum IgG, IgM, IgA, IgG1, 2, 3, by ELISA | | Pre and post lung transplant | | GOLD Stage 4, FEV1<30% post-bronchodilator |
| *Noda, 1989* | CS, SC | 13 |  | | Serum secretory IgA by ELISA | |  | | Chronic bronchitis, American Thoracic Society |
| *Oh 2024** | AD | 7223 |  | | IgG2, unspecified | |  | | COPD, unspecified |
| *O'Keeffe 1991** | CS, SC | 58 | 125 | | Serum IgG,IgG1, IgG2, IgG3,IgG4 by rate nephelemetry | | Healthy controls | | COPD clinical and spirometry (not specified) |
| *Olusi 1983* | CS, SC | 24 | 680 | | Serum IgG, IgA, and IgE by radial immunodiffusion (Mancini) | | Healthy controls | | COPD based on clinical presentation, radiological studies and vital capacity |
| *Orfanakis 1973** | R, SC | 87 |  | | IgA, IgG, IgM by radial immunodiffusion, nasal IgA by an adaptation of the technique from Lowry and associates | |  | | Unspecified |
| *Palikhe 2023* | P, SC | 51 |  | | Serum IgA, IgG, IgM by turbid metric process (Alberta Precision Laboratories) | |  | | COPD diagnosed by a respirologist |
| *Paparo 1994* | P, SC | 30 |  | | Serum secretory IgA by radial immunodiffusion | |  | | Chronic bronchitis defined by the ATS 1987 |
| *Pla 2021** | P, MC | 295 |  | | Serum IgG, by unspecified method | | Exacerbators (>=2 exacerbations in year) vs non-exacerbators | | COPD, not specified (post ronch FEV1 10-96% among participants) |
| *Putcha 2017* | R, AD | 1049 |  | | Serum IgA by the Myriad RBM biomarker discovery platform | | Normal vs low IgA | | Post-bronchodilator FEV1/FVC < 70% |
| *Peng 2021** | RCT, SC | 115 |  | | Serum IgA, IgM, IgG using the TIIA method | | Enteral nutrition vs none | | COPD, FEV1 < 50%, FEV1/FVC < 70% |
| *Polosukhin 2011** | CS, SC | 36 |  | | Serum IgA and secretory IgA by ELISA | |  | | COPD by GOLD, not speciifed |
| *Quavordt 2001* | CS, SC | 33 | 67 | | Serum IgG, IgA, IgM, IgG1, 2, 3, 4 by radial immunodiffusion | | Controls | | Chronic bronchitis, defined by the ATS (FEV1 < 80%) |
| *Southworth, 2020** | CS, SC | 41 |  | | BAL IgA, IgM, IgG1, IgG2 by ELISA | | Eosinophil high vs eosinophil low COPD | | FEV1/FVC < 70% |
| *Tejwani 2022** | P, SC | 211 |  | | BAL and serum IgG, IgA by ELISA | |  | | Not specified |
| *Turnbull, 1977* | CS, SC | 26 |  | | Sputum and serum IgG, IgA, IgM, IgE by single radial diffusion | |  | | Chronic bronchitis defined as persistent cough and sputum for 3 months in at least 2 consecutive years, at least one period of illness which had kept patient from normal activities for at least 3 weeks in 2 successive years, and reversibility of airways obstruction of not mre than 20% of initial FEV1 after 1 or 2 inhalations of salbutamol, and no evidence of pulmonary TB, bronchiectasis or bronchial carcinoma |
| *Vogt 2021* | MC, post-hock analysis of RCT | 178 |  | | Serum IgG and subclasses were measured on an Image analyser using Sanquin reagents and a Sanquin calibrator. San quin standard was used and values were traceable to the WHO 67/97 standard. IgA and subclasses measurement was performed with a commercially available liquid latex reagent kit using the Beckman Image 800 (Beckman Coulter) machine and Sanquin reagents. | | MBL deficiency vs not | | COPD as defined in REDUCE study (GOLD criteria) |
| *Zinneman 1980** | SC, CS | 91 |  | | Serum, salivary, sputum IgA, IgM, IgG by single radial immune diffusion | | Alpha 1 antitrypsin deficiency vs not | | FEV1 < 60% predicted |
|  |  |  |  | |  | |  | |  |
|  |  | | |  | |  | |  |  |
|  |  | | |  | |  | |  |  |

*not included in meta-analysis

**Supplemental Table 2.** Study Location

| **Country of Study** | **Number of studies (%)** |
| --- | --- |
| Canada | 6 (12.24) |
| China | 6 (12.24) |
| Denmark | 1 (2.04) |
| Egypt | 1 (2.04) |
| India | 5 (10.20) |
| Ireland | 1 (2.04) |
| Japan | 1 (2.04) |
| Mexico | 1 (2.04) |
| Nigeria | 1 (2.04) |
| Norway | 1 (2.04) |
| Scotland | 1 (2.04) |
| Sweden | 1 (2.04) |
| Switzerland | 1 (2.04) |
| Turkey | 1 (2.04) |
| United Kingdom | 1 (2.04) |
| United States of America | 17 (34.69) |
| Vietnam | 2 (4.08) |
| Yugoslavia | 1 (2.04) |

**Supplemental Table 3**. Summary of cut-off values used to defined Ig deficiency

|  | Immunoglobulin Class | | | | | | |
| --- | --- | --- | --- | --- | --- | --- | --- |
|  | IgG (g/L) | IgA (g/L) | IgM (g/L) | IgG Subclasses | | | |
|  |  |  |  | IgG1 | IgG2 | IgG3 | IgG4 |
| Alotaibi | < 7 |  |  |  |  |  |  |
| Burnim 2024 | < 7 | < 0.7 * |  |  |  |  |  |
| Filho 2018 |  |  |  | < 2.80 | < 1.15 | <0.24 | <0.052 |
| Filho 2020 | < 7 |  |  |  |  |  |  |
| Hai 2024 | < 7.68 | <0.82 | <0.6 * | <3.82 | <2.42 | <0.22 | <0.04 |
| Helela 2022 | < 7.09 |  |  | <2.80 | <1.15 | <0.24 | <0.052 |
| Holm 2020 | < 6.1 |  |  |  |  |  |  |
| Lee 2022 |  |  |  | <2.80 | <1.15 | <0.24 | <0.052 |
| Palikhe 2023 | < 6.94 | <0.7 * | <0.6 |  |  |  |  |
| Paul |  | <0.7 |  |  |  |  |  |
| Vogt 2021 | < 7.5 | IgA1 < 0.6 IgA2 < 0.06 |  | < 4.9 | < 1.5 | < 0.2 | < 0.08 |

* no direct comparison between COPD with low Ig of this class compared to COPD with normal Ig of this class

**Supplemental Table 4**. Risk of bias assessment

| **Study Name** | **Bias due to confounding** | **Bias in selection of participants** | **Bias of classification of interventions** | **Bias due to deviations from intended interventions** | **Bias due to missing data** | **Bias in measuring outcomes** | **Bias in the selection of the reported result** | **Overall bias** |
| --- | --- | --- | --- | --- | --- | --- | --- | --- |
| Ablin, 1973 | serious | moderate | n/a | n/a | low | moderate | moderate | moderate |
| Akhtar, 2000 | serious | serious | moderate | n/a | moderate | serious | serious | moderate |
| Alotaibi, 2021 | low | low | low | n/a | low | moderate | moderate | low |
| BaTa, 2022 | low | moderate | moderate | moderate | moderate | low | low | low |
| Berber, 1995 | serious | serious | serious | n/a | serious | moderate | serious | serious |
| Bhave 1988 | moderate | moderate | moderate | moderate | serious | moderate | moderate | moderate |
| Biegel, 1968 | moderate | serious | moderate | n/a | serious | serious | moderate | moderate |
| Burnim, 2024 | low | moderate | low | n/a | low | moderate | low | low |
| Cass, 2021* | serious | moderate | low | n/a | serious | low | low | moderate |
| Chauhan, 1990 | serious | serious | moderate | n/a | moderate | moderate | moderate | moderate |
| Cvoriscec, 1989 | serious | moderate | serious | low | serious | moderate | moderate | moderate |
| Dalvi, 1990 | serious | moderate | moderate | n/a | moderate | low | moderate | moderate |
| Dasgupta *1998* | serious | moderate | low | n/a | moderate | moderate | moderate | moderate |
| Du, 2015 | moderate | moderate | moderate | n/a | moderate | low | moderate | moderate |
| Falk, 1971 | serious | moderate | moderate | n/a | moderate | serious | moderate | moderate |
| Falk, 1972 | serious | moderate | low | n/a | serious | moderate | low | moderate |
| Hai, 2024 | low | moderate | low | n/a | low | low | low | low |
| Harbitz, 1980 | serious | moderate | moderate | n/a | moderate | serious | moderate | moderate |
| He 2023 | serious | moderate | low | n/a | moderate | low | low | low |
| Helala, 2022 | low | low | low | low | moderate | low | moderate | low |
| Holm, 2020 | low | moderate | low | low | low | low | low | low |
| Ho, 2024 | serious | moderate | low | n/a | moderate | moderate | moderate | moderate |
| Ji, 2023 | low | low | low | n/a | serious | low | low | low |
| Karnak, 2001 | serious | moderate | low | n/a | moderate | moderate | low | moderate |
| Klaustermeyer, 1992 | moderate | moderate | moderate | n/a | moderate | moderate | moderate | moderate |
| Lee, 2022 | moderate | low | low | low | moderate | low | low | low |
| Leitao Filho, 2018 | low | low | low | low | low | moderate | moderate | low |
| Leitao Filho, 2020 | low | low | low | low | low | moderate | low | low |
| Li, 2006 | serious | moderate | low | low | moderate | moderate | moderate | moderate |
| Liu, 2020 | serious | low | low | n/a | moderate | moderate | moderate | moderate |
| McCullagh, 2017 | moderate | low | low | n/a | low | low | moderate | low |
| McQuiston, 2022 | moderate | moderate | moderate | n/a | serious | serious | moderate | moderate |
| Noda, 1989 | serious | serious | moderate | n/a | moderate | serious | moderate | serious |
| Oh, 2024 | moderate | moderate | moderate | n/a | moderate | moderate | moderate | moderate |
| O'Keeffe 1991 | serious | moderate | low | n/a | serious | moderate | low | moderate |
| Olusi, 1983 | moderate | moderate | moderate | n/a | moderate | moderate | low | moderate |
| Orfanakis 1973 | moderate | serious | low | n/a | moderate | moderate | moderate | moderate |
| Palikhe 2023 | moderate | low | low | n/a | moderate | low | moderate | moderate |
| Paparo, 1994 | serious | moderate | moderate | moderate | serious | moderate | moderate | moderate |
| Pla, 2021 | serious | moderate | moderate | n/a | moderate | moderate | moderate | moderate |
| Putcha, 2017 | moderate | low | low | n/a | moderate | low | moderate | low |
| Peng, 2021 | moderate | low | low | n/a | low | low | moderate | low |
| Polosukhin, 2011 | serious | low | low | n/a | moderate | low | serious | moderate |
| Quavordt, 2001 | low | low | low | n/a | moderate | low | low | moderate |
| Southworth, 2020 | serious | low | moderate | n/a | serious | moderate | moderate | moderate |
| Tejwani, 2022 | moderate | moderate | moderate | n/a | moderate | moderate | moderate | moderate |
| Turnbull, 1977 | moderate | moderate | moderate | n/a | serious | moderate | moderate | moderate |
| Vogt, 2021 | low | low | low | n/a | moderate | low | low | low |
| Zinneman, 1980 | serious | moderate | moderate | n/a | moderate | moderate | moderate | moderate |
